# Wrist-to-Tibia/Shoe Inertial Measurement Results Translation Using Neural Networks

**DOI:** 10.3390/s24010293

**Published:** 2024-01-03

**Authors:** Marcin Kolakowski, Vitomir Djaja-Josko, Jerzy Kolakowski, Jacek Cichocki

**Affiliations:** Institute of Radioelectronics and Multimedia Technology, Warsaw University of Technology, 00-661 Warsaw, Poland

**Keywords:** gait analysis, neural networks, machine learning, signal translation, autoencoders

## Abstract

Most of the established gait evaluation methods use inertial sensors mounted in the lower limb area (tibias, ankles, shoes). Such sensor placement gives good results in laboratory conditions but is hard to apply in everyday scenarios due to the sensors’ fragility and the user’s comfort. The paper presents an algorithm that enables translation of the inertial signal measurements (acceleration and angular velocity) registered with a wrist-worn sensor to signals, which would be obtained if the sensor was worn on a tibia or a shoe. Four different neural network architectures are considered for that purpose: Dense and CNN autoencoders, a CNN-LSTM hybrid, and a U-Net-based model. The performed experiments have shown that the CNN autoencoder and U-Net can be successfully applied for inertial signal translation purposes. Estimating gait parameters based on the translated signals yielded similar results to those obtained based on shoe-sensor signals.

## 1. Introduction

Recently, gait analysis has become an essential tool in healthcare, sports, and fitness. It is especially prevalent in elderly care as the gait and balance deficits increase with age. The gait analysis helps to estimate the risk of falls and screen for frailty [1]. Gait-related parameters are also commonly used for intrinsic capacity (IC) and functional ability evaluation [2] as they correspond to the performance of the older adults in the IC locomotion domain.

Besides IC, there are several geriatric assessment tools based on gait speed and dynamic balance. The most renowned tests measure the time it takes to cover a given distance in various conditions. The most straightforward, the 10-meter walk test, assesses walking speed over a short distance [3]. More complex solutions assume following a defined path, e.g., walking a figure eight shape [4] or moving along a narrow path between parallel lines [5]. Others require passing over obstacles or keeping a straight walk while turning the head to the right or left or tipping the head up and down [6].

As human movement can be accurately measured using inertial sensors, automatization of such evaluation procedures with wearable sensors seems an obvious solution. The application of inertial sensors to geriatric gait assessment is presented in many publications. In the survey [7], inertial sensor-based frailty and fall risk evaluation methods are compared. They differ in the number of sensors used and their position. Six inertial measurement units (IMUs) were even used in the most extensive test setups. The sensors are typically attached to feet, instep, heels, the chest, the sternum, trunks, or the tibia or combinations of these locations.

Most tests are performed in clinical conditions where numerous sensors on different body parts can be easily placed. Unfortunately, such an experimental setup is hard to recreate in everyday living conditions. In such applications, the sensors should fulfill several requirements: they should be easy to operate and charge and should not cause the user any significant discomfort while putting them on or taking them off. Otherwise, such solutions might not be accepted by the older adults. Despite the problems mentioned above, great hope is being placed in such solutions as monitoring the gait of older adults during everyday activities would provide data better reflecting their overall health state [8,9].

Another important area of gait analysis application is related to sports, fitness, and rehabilitation. In these cases, most of the tests described in the literature utilize sensors mounted in the lower limb area. In the comprehensive review [10] of running gait analysis methods, out of 170 analyzed works, over 100 relied on measurements performed in the lower limb area (tibia, shoe, insoles), whereas a mere 2 works obtained data with a wrist-worn sensor.

Generally, regardless of the domain, most gait evaluation tests with inertial measurement units are performed using shoe or tibia-mounted sensors. Although giving accurate results in laboratory environments, such sensors are unsuitable for daily monitoring. Shoe-based sensors are fragile and require the same pair of shoes to be worn all the time. Tibia-mounted sensors are usually uncomfortable, and their orientation might shift if not tightly attached. A much better solution would be to use inertial measurement units embedded in smartwatches or smart bands worn by the users.

The motivation for the following study is to find a method that would enable the translation of signals recorded by an inertial sensor worn on the wrist to signals that would be recorded by sensors placed in the lower limb area: tibia and foot. The main contributions of this study are the following:

- We analyzed four different neural network architectures for wrist-to-tibia and wrist-to-shoe translation of inertial signals (acceleration and angular velocity),
- We gathered a dataset comprising inertial measurements registered using the wrist, tibia, and shoe-worn sensors collected over several hours of walking.
- Our experiments have proved that the U-Net-based model and the CNN autoencoder can be successfully applied to solve IMU signal translation problems.

The structure of the rest of the paper is as follows. Section 2 describes the current state of the art concerning neural-network-based signal translation methods. The problem and the proposed inertial signal translation model are outlined in Section 3 and Section 4, respectively. The experiments and results are presented in Section 5. Section 6 concludes the paper.

## 2. Related Work

Signal translation using neural networks is a vibrant field of research. Most of the articles written on the matter concern image-to-image translation [11] including style transfer [12] and generating semantic maps [13]. The research described in the literature is mainly concerned with the conversion of photos or videos. The works that address translating signals obtained through means other than visual are much less common. In this section, we analyze works that employ machine learning (ML)-based signal translation methods to solve relevant problems, converting the signals to those obtained using different sensors or under different conditions.

There are different reasons to apply signal translation. In most cases, its purpose is to transform a signal into another domain, which enables more efficient data processing, for example, by using ML models trained on data gathered using a different sensor [14] or to change the signal representation to one which is widely known and easier to interpret by human experts [15]. Most of the signal translation methods use one of two network architectures: autoencoders [16] or generative adversarial networks (GANs) [17]. The employed models range from simple feed-forward networks to sophisticated architectures, including several convolutional and LSTM (long short-term memory) layers.

The method proposed in [14] translates range-Doppler maps obtained using a newer version of an ultra-wideband (UWB) radar to a domain associated with its older counterpart. The main goal is to enable the use of ML models trained on data gathered using the previous sensor version. The authors propose a novel Sig2Sig architecture that builds upon the well-known Pix2Pix model [18]. The model consists of a U-Net-shaped generator [19] with squeeze and excitation blocks used for information compression. The generator is followed by a multi-channel attention selection module, which allows the model to obtain uncertainty maps for calculating losses, in which more important pixels are taken with greater consideration. The experiments have shown that the translated signals can be successfully used with previously trained ML models. The performance classification model was very close to that obtained using the data collected with the older sensor.

Signal translation can solve a similar problem when the results returned by the sensor are harder to interpret by human experts than their standard counterparts. In [15], an adapted version of the CycleGAN model [20] is proposed to transform raw ECG (electrocardiography) measurements from a novel 11-channel contactless capacitive sensor into the standard output of 12-channel wet electrode ECG. As the original CycleGAN architecture was intended for 2D image processing, the authors propose a simplified architecture in which the convolution and residual blocks in both generator and discriminator are omitted (the tests performed by the authors have shown that their presence slows the model’s performance without significantly enhancing its performance). The model is compared against three typical architectures: MLP, PIX2PIX, and LSTM. For most leads, the obtained ECG measurements are more accurate. The quality of the resulting ECG measurements enables their use by medical professionals for heart health assessment.

A similar problem is solved in [21], where the seismocardiogram signals are translated into the domain of ECG distance transform. The authors propose the SeismoNet architecture, which is a modification of the U-Net model [19]. The U-Net model is prepended with a convoluted ensemble averaging block consisting of several convolutional layers, whose purpose is to reduce the signal jitter. At the U-Net output, a denoising block is appended. The obtained signals’ quality allows for high-accuracy heart rate parameter estimation.

Another blood-flow-related application is presented in [22], where photoplethysmogram (PPG) signals are converted to blood pressure (BP) waveforms, which are easily interpretable by doctors. The proposed method uses the generalized regression neural network (GRNN) [23] as a basis. The GRNN consists of four subsequent layers: input, pattern, summation, and output. The pattern layer uses a radial basis activation function. The signal is processed in the following manner: First, a single period of the PPG is encoded into N harmonics. The harmonics are fed into the GRNN model, which converts them into N harmonics of the BP signal, which are then decoded into the BP waveform signal. The network achieved high signal reconstruction accuracy, conforming with restrictive medical device requirements.

Signal translation has been applied in processing medical images. In [24], a modification of the Cycle-GAN network [20] called Cycle-MedGAN is used to translate positron emission-computed tomography (PET) to computer tomography (CT). Cycle-MedGAN introduces novel non-adversarial loss functions, which are calculated using a pre-trained feature extractor. The new loss function is supposed to capture the perceptual aspects of the generated image quality, which pixel-wise loss analysis does not cover sufficiently. The experiments showed that the proposed architecture performs better in a PET-CT translation scenario than the original Cycle-GAN network. A similar study, where CT images are converted to magnetic resonance (MR), is presented in [25]. The authors propose a GAN-based translation model in which an additional U-Net-like segmentation network is used to improve the semantic content consistency between the generated images. The demonstrated results proved the proposed model’s effectiveness.

Signal translation also has uses in brain activity monitoring, where it is used to interpret brain signals. In [26], a novel MSATNet (Multi-Scale Activity Transition Network) model is proposed to mitigate the translation problems that occur in convolutional neural networks (CNNs). The proposed network consists of several subsequent activity structure blocks (including multiple convolutional layers) followed by pooling layers. The features returned by each block are processed with activity transition blocks (composed of LSTMs) and then are concatenated prior to the final fully connected layer. The proposed model is used for electroencephalogram (EEG) decoding. The results have shown that the proposed solution is superior to the analyzed counterparts.

A different application of brain signal translation is presented in [27], where 3D continuous hand movement is predicted based on the time-frequency features of electrocorticography signals. For this purpose, the authors consider five network architectures:

- CNN-FC (fully connected);
- CNN-LSTM, outputting a single sample;
- CNN-LSTM, outputting a part of the signal trajectory;
- CNN-LSTM-FC, where the outputs of the LSTM layers are subject to an additional time-based convolution prior being fed to the fully connected layer;
- CNN-FC, where 3D convolutions are applied to process whole signal sequences at the same time.

The results of the experimental evaluation are not consistent, as some of the models perform better for some axes than others. The best overall performance was achieved using the CNN-LSTM model, translating a part of the signal trajectory.

Signal translation can also be used in other scenarios, e.g., assessing structural damage suffered by large-scale objects. In [28], a model based on the transformer architecture [29] is used to translate acceleration measured by sensors mounted on a bridge to live load displacement caused by passing cars. The model consists of two modules: the encoder and the decoder. The layers used in the modules include self-attention followed by fully connected and normalization sub-layers. The decoder layers include an additional encoder–decoder attention sub-layer, which allows the self-attention mechanism to be applied over the encoder output. The tests performed at a highway showed that the displacement estimate obtained with the proposed model is much more accurate than in the case of the alternative free vibration method.

Gait-based user authentication is another area where converting the signals may be required. In such methods, the inertial sensors worn by the user are used to analyze their gait and movement patterns and extract their unique features, which allow for user identification and subsequent authentication, making the wearable systems more secure. One of the main problems of such solutions is the high dependence between the sensor readings and its location. Calibrating the authentication for one sensor location might result in misidentification when the sensor is worn differently.

In [30], the authors propose to use an ML model, which translates the signals from a device worn in an unusual location to a standard one. The proposed model is an autoencoder including several dense layers and is trained in a supervised setting. The model is finely tuned to the particular users using the transfer-learning approach. The model was tested for different sensor location translation scenarios (chest to waist, head, shin, thigh, and upper arm). The results showed that the translated signals can be successfully used for authentication key generation. The problem of user identification in the case of variable sensor location scenarios is also addressed in [31], where a method based on a unified autoencoder framework is used. The AE directly extracts discriminative features of the user’s gait and simplifies the whole authentication algorithm. To consider the geometry of the data, the authors propose to use the spectral angle distance instead of the traditional mean squared error (MSE).

Signal translation can also find its application in gait parameter analysis for medical purposes. For example, the gait parameters may be calculated by analyzing a video feed of a walking person. In such algorithms, the subsequent frames are analyzed to obtain the user’s pose and location of limbs. In [32], a model of stacked progressive autoencoders (SPAEs) is employed to convert the gait energy images (GEIs) registered for people dressed in untypical, complicated clothes, e.g., down jacket or long coat, to images of people in typical clothing. The model consists of two stacked autoencoders that aim to map the GEIs of persons in unconventional clothing to typical conditions while keeping the typical GEIs unchanged.

A more traditional way to analyze gait parameters is to use inertial sensors. In the literature, many methods and ML models require the sensors to be mounted on shoes or user’s tibias. In order to apply these methods in scenarios where the user wears a smartwatch, the obtained signal must be translated. In [33], a neural network is used to translate the readings of the wrist-worn accelerometer to readings of accelerometers mounted on each of the ankles. The proposed network model consists of three types of layers: CNN, LSTM, and dense. The model takes a sequence of acceleration samples from a wrist sensor and outputs six samples corresponding to readings in three axes of right and left ankle-worn sensors. The obtained results are superior compared to a simple feed-forward network. Although the model gives satisfactory results, it comes with certain significant limitations. First, as the model outputs samples instead of whole sequences, translating longer signals requires repeatedly calling the model using the sliding window technique. The model also has high computational complexity as the layers have nearly 12 million trainable parameters. Such complexity and the use of slow LSTM lowers its applicability to more powerful units and prevents it from being implemented directly in the wearables. Additionally, the network translates only the acceleration measurements. It does not consider the angular velocity, which allows for accurate evaluation of gait parameters due to exhibiting significant changes even when the movements are less intensive.

## 3. Inertial Measurements at Different Locations

**Figure 1:**
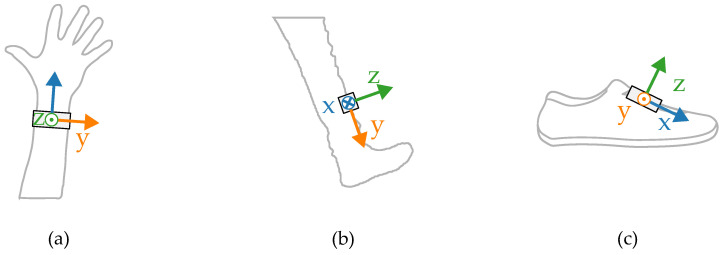
Sensor placement during the initial tests on (**a**) a wrist, (**b**) tibia, (**c**) shoe.

Most wearable gait evaluation systems utilize measurements from IMUs including a tri-axial accelerometer and a tri-axial gyroscope. The amplitudes and shapes of the signals are highly dependent on how and where the sensor is worn. In order to illustrate the differences, a small set of inertial measurements registered concurrently in three locations was gathered. The placements of the sensors during the experiments are presented in Figure 1.

**Figure 2:**
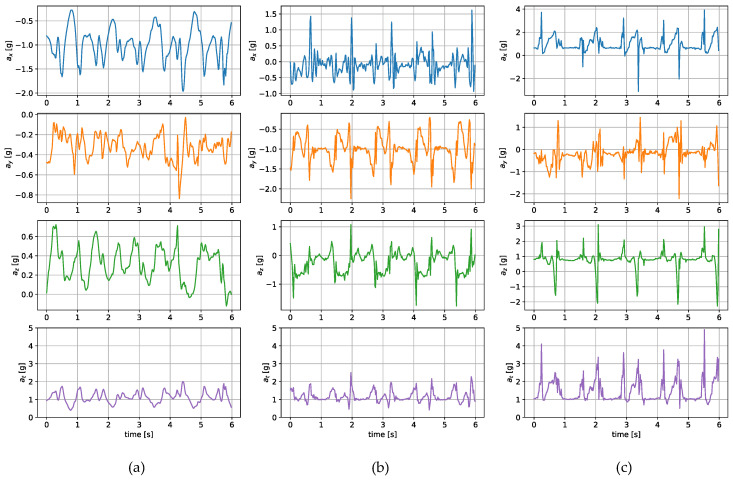
Exemplary acceleration in *x*, *y*, *z* axes and its resultant value measured for a sensor placed on (**a**) a wrist, (**b**) a tibia, (**c**) a shoe.

**Figure 3:**
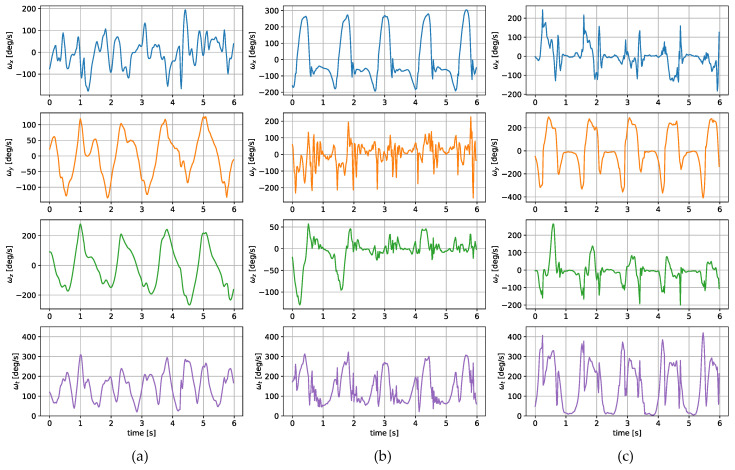
Exemplary angular velocity around *x*, *y*, *z* axes and its total value measured for a sensor placed on (**a**) a wrist, (**b**) a tibia, (**c**) a shoe.

The measurements were taken with the sensors attached in three places: to a wrist, to a tibia, and mounted on a shoe. The direction of the axes differ between the sensor placements. Therefore, the measurements registered with those sensors are not easily interchangeable. Exemplary six-second acceleration and angular velocity sequences recorded for a walking person are presented in Figure 2 and Figure 3, respectively.

In the case of both acceleration and angular velocity, the signals differ significantly. The results gathered in the same sensor axes differ due to different sensor orientations. In the case of acceleration measurements, both the tibia and shoe-mounted sensor return signals with higher amplitudes. The waveforms are also sharper and include distinct short peaks, making it easier to perform gait timing measurements. The most evident peaks are in the shoe-sensor signals due to the high acceleration measured during the heel strike.

The angular velocity measured does not significantly differ in terms of the signal amplitude. However, the difference in waveform shapes is significant. For the wrist sensor, the waveforms for *y z* axes and the resultant values are sine-like, which makes it hard to distinguish the stride phases, and only steps can be efficiently counted. In the case of the tibia and shoe sensors, the shapes are more distinct, and we can observe positive and negative peaks in the signal, which correspond to the boundaries between the stance and swing phases.

The different shapes of the waveforms make it impossible to find an analytical solution, allowing for a straightforward translation between them. In this case, the most viable option is to train an ML algorithm to establish their relationships and dependencies.

## 4. Translation Algorithm

The main aim of the translation algorithm is to convert the signals obtained with a wrist-worn sensor to signals that would be observed with a tibia or a shoe-mounted device. In Section 3, it was established that due to the complex relationships between those signals, it would be nearly impossible to find an analytical solution for a straightforward conversion, and ML should be used. The employed ML solution should fulfill two conditions. First, it should be relatively simple to ensure its deployability to the sensor device. Second, it should translate a whole piece of the signal trajectory at one time, as repeatably running the model using the sliding window technique is energy-inefficient.

### 4.1. General Idea

**Figure 4:**
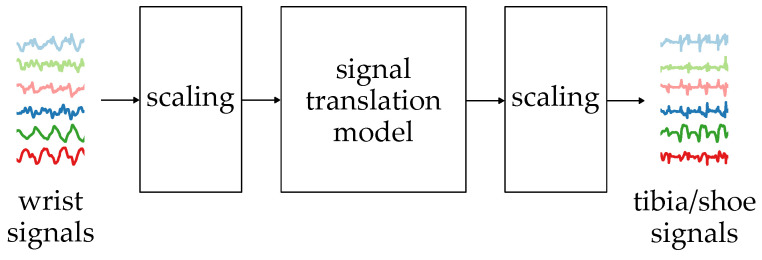
The general idea of the signal translation algorithm.

The general idea of the proposed signal translation algorithm is presented in Figure 4.

The signal translation is performed using a neural network which takes six sequences measured with the wrist-worn inertial sensors. The sequences are acceleration ($a_{x}$, $a_{y}$, $a_{z}$) and angular velocity ($\omega_{x}$, $\omega_{y}$, $\omega_{z}$) measured in three axes. The sequences contain 256 samples, which, in the case of a 50 Hz sampling rate, correspond to 5.12 s, a period in which at least two full strides should be observed. The measurement results are scaled so the values are in the 0–1 range.

The neural network’s output is six translated sequences, which are then scaled to their standard measurement units. The results can then be processed with algorithms or ML models relying on lower limb inertial measurement results.

### 4.2. Neural Network Architectures

The works referenced in Section 2 include the description of several neural network architectures used for signal translation. In our work, we tested four different neural network architectures, some of which are similar to those that yield satisfying results in the referenced works. The adopted architectures are not overly complicated, so they could be implemented directly on wearables.

In the study, four different architectures were considered:

- a dense fully connected autoencoder (DNN—dense neural network),
- a convolutional autoencoder,
- a CNN-LSTM network,
- a U-Net-based network.

**Figure 5:**
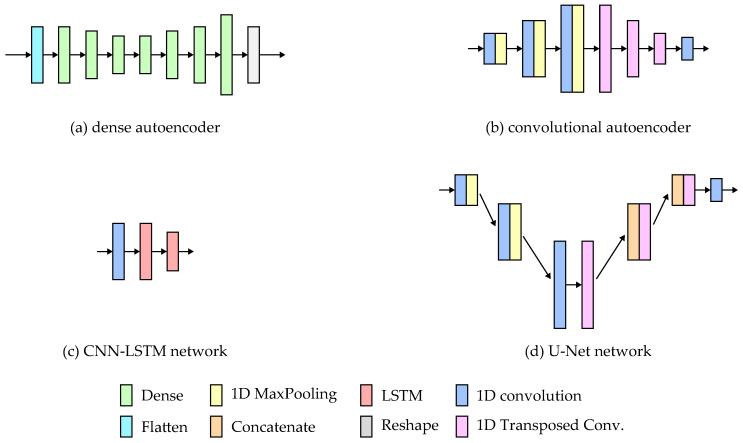
Architectures of the tested neural network models.

The simplified architectures of the models are presented in Figure 5.

The autoencoder network consists of two parts: the encoder and the decoder. In the case of the fully connected dense autoencoder, both parts comprise dense layers. The input sequences are flattened into a one-dimensional vector, which the encoder processes to extract features and represent the input in the latent dimension. The decoder layers are responsible for signal reconstruction. The network’s last layer reshapes the output into six 256-point sequences. The implementation details of the network are presented in Table 1. For all of the architectures, the layer parameters were chosen manually based on initial tests.

The Convolutional autoencoder has a similar structure to the fully connected one. The only difference is that the dense layers were replaced with 1D convolutions in the encoder and 1D transposed convolutions in the decoder. The layer details are presented in Table 2.

The CNN-LSTM network used in the study is based on the solution presented in [27], where such a solution yielded good results for hand motion prediction based on brain signals. The network consists of a single 1D convolution layer, which extracts features from the inertial signals. The temporal relations between the features were analyzed using two LSTM layers. The network parameters are stored in Table 3.

The U-Net architecture is typically used for image reconstruction in image segmentation tasks [19]. The network has a similar structure to the autoencoder, where the signal is encoded into the latent dimension and then reconstructed. U-Net employs additional skip connections between layers, which allow the signals’ high-level features to be passed to the reconstructing layers. The features are concatenated with the previous reconstruction layer output and fed to the next layer. Such an approach allows the network to take into consideration both high- and low-level features while rebuilding the signal. The details of the implemented layers are presented in Table 4.

## 5. Experiments

### 5.1. Gait Sensors

**Figure 6:**
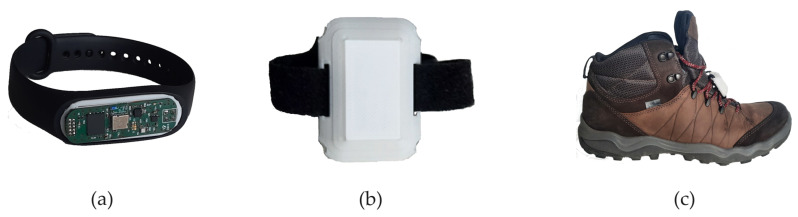
Sensors used in the study: (**a**) the wrist-worn sensor, (**b**) the tibia-worn sensor, (**c**) the shoe-mounted sensor.

Photographs of the gait sensors used in the study are presented in Figure 6

The gait sensor used in the study is a small wearable device intended to estimate gait parameters. The sensor is controlled with a BLE-enabled nRF52833 Nordic Semiconductor microcontroller and includes one Bosch Sensortec BMI270 Inertial Measurement Unit and one BMP250 barometer. In the study, only the outputs of the IMU were used. The sensors were placed in custom 3D-printed cases. The wrist sensor (Figure 6a) used a smart band strap compatible case. For the tibia (Figure 6b) and shoe sensors (Figure 6c), a special case that could be attached using Velcro straps or strings was designed.

The IMU used in the sensor was set to measure the acceleration and angular velocity in three axes with a 50 Hz frequency. The 50 Hz sampling frequency is lower than recommended for gait analysis (at least 120 Hz [34]) but keeps energy usage at acceptable levels. The results are stored in the device’s internal memory and then are copied to the tablet over a USB connection. For the experiment, the device’s firmware was updated to enable a synchronized start of measurements after BLE advertisement packet reception.

### 5.2. Datasets and Training

The process of model training depended on its architecture. The feature detector parts of the autoencoder models and U-Net were pre-trained using a large dataset of wrist-sensor measurements in an unsupervised learning scenario where the wrist signals were both inputs and labels. The feature extraction layers were then frozen, and only the reconstruction layers were trained using the custom wrist-to-tibia and wrist-to-shoe translation datasets. The CNN-LSTM model was directly trained using the translation dataset. The wrist-only measurements dataset comprised 7200 samples (corresponding to about 10 h of constant walking). The results were collected during the initial tests of the gait sensor both in inside and outside scenarios.

The wrist-to-tibia and wrist-to-shoe translation datasets included 1000 samples each, which corresponded to roughly 85 min of constant walking. The datasets, for the most part, were registered during different measurement sessions, as all three sensors were rarely used simultaneously. During the training of the models, the datasets were divided into three parts: training, validation, and testing, with a 70/15/15 proportion. The proposed models were implemented using TensorFlow [35]. The mean squared error loss function and the Adam optimizer with 1 × 10${}^{-3}$ learning rate were used for all of the models. The encoders and the U-Net network were finely tuned by unfreezing the encoder part and training the model with a lower 1 × 10${}^{-4}$ learning rate.

### 5.3. Model Evaluation

**Figure 7:**
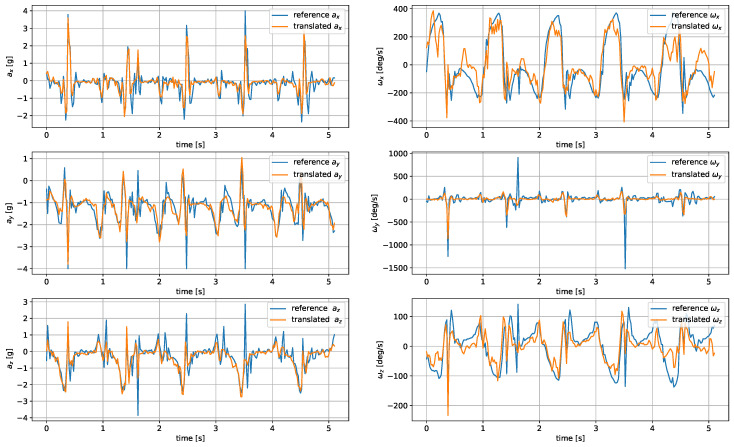
Translated tibia-worn sensor signals obtained using the U-Net network.

**Figure 8:**
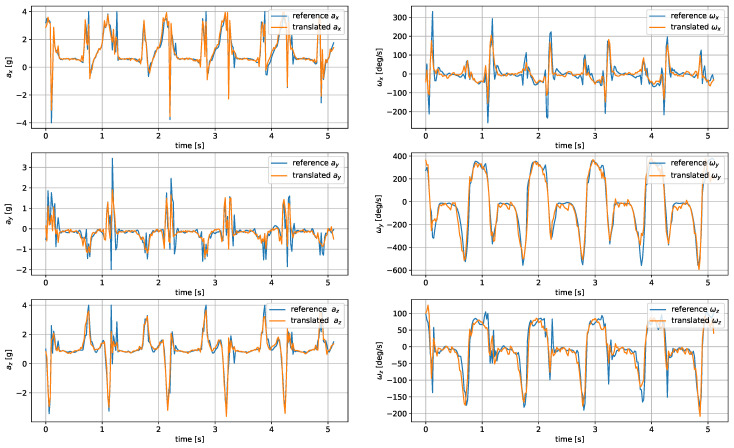
Translated shoe-worn sensor signals obtained using the CNN autoencoder.

The model was evaluated based on the mean average error and root mean square of the reconstructed signals. The comparison of the metrics for the analyzed architectures and wrist–tibia and wrist–shoe scenarios are presented in Table 5 and Table 6, respectively. The exemplary reconstruction is shown in Figure 7 and Figure 8.

The most accurate translation between the wrist and tibia inertial measurements was achieved by U-Net, closely followed by the CNN autoencoder. The LSTM-generated signals were less accurate, as the applied architecture had problems reproducing the sharp peaks occurring during heel strikes. The worst accuracy was observed for the DNN network.

For shoe-sensor measurements, the best translation algorithm was the CNN autoencoder, followed by DNN and U-Net. The problems with proper replication of signal spikes by the LSTM network were more noticeable due to their higher amplitudes compared to the tibia-registered signals.

In both wrist-to-tibia and wrist-to-shoe translation, the biggest challenge was properly reconstructing the signal peaks. Such difficulties might result from a relatively low 50 Hz IMU sampling rate. It might pose a problem in threshold-based step detection methods. However, it should not be a critical issue for gait investigation methods depending on peak detection and analysis of periods between them.

### 5.4. Gait Parameter Estimation

The translated data were used to calculate the user’s gait frequency. Gait frequency can be directly associated with gait speed, which is one of the main parameters used to evaluate the IC in the locomotion domain. The results were compared to those obtained based on the acceleration measured with the shoe-mounted sensor. For this purpose, a short test, during which a person walked along a straight 10 m line, was executed. During the test, the person walked the path twice (there and back), and the time taken was measured.

**Figure 9:**
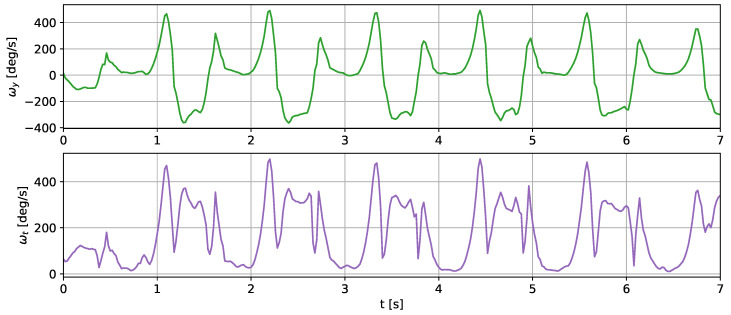
Angular velocity in the *y* axis and the resultant value registered using the shoe sensor during a 10 m walk. The *y*-component of the angular velocity was inverted to match the orientation assumed in [36].

For this test, we used the gait frequency estimation method described in [36]. In this approach, the gait parameters are estimated through analysis of the *y*-component of the angular velocity and its signal vector magnitude. The *y*-component and the vector magnitude were filtered using a Butterworth low-pass filter. In [36], a 12 Hz cut-off frequency was used. In our test, we used a narrower filter of 5 Hz to remove possible noise resulting from the signal reconstruction. The *y*-component and the resultant value of the angular velocity measured with the shoe sensor are presented in Figure 9.

**Figure 10:**
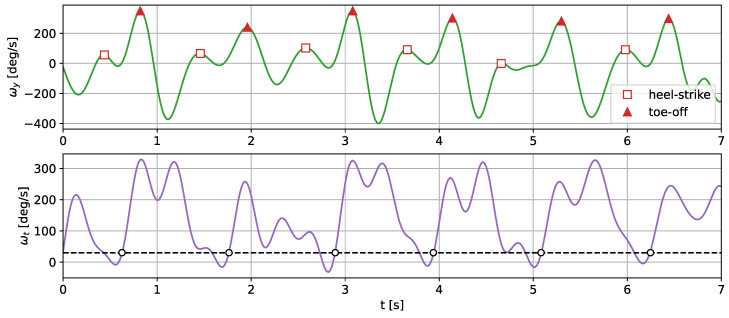
The filtered *y*-component and the resultant value of the angular velocity translated with the CNN autoencoder. The detected toe-off moments are marked with a triangle and the heel strikes with diamonds.

The gait temporal parameters were established through the detection of two types of characteristic gait events: toe-off points and heel strikes. Both of those events correspond to peaks in the *y* axis angular velocity. In order to determine which peak corresponds to which category, the edge of the resultant angular velocity was analyzed. The peaks, closer to a moment when the signal passes a threshold, correspond to the toe-off events. The detected toe-off and heel strike events are marked on the translated signal and are presented in Figure 10.

The heel strikes and toe-off points divide the individual strides into the swing and stance phases, which enables a more detailed gait analysis. The comparison of the temporal gait parameters calculated based on the actual shoe-measured angular velocity and the translated signals is presented in Table 7.

The calculated mean stride and gait durations are close to each other—the difference between the obtained values is 28 and 14 ms, respectively. There was an observable difference in the stride phase length estimation. The differences were 80 and 44 ms.

## 6. Conclusions

The paper presents and evaluates the concept of inertial measurement translation between different sensor locations. For this purpose, four different neural network architectures were implemented and tested on a custom dataset. The analyzed architectures included FC and CNN autoencoders, a CNN-LSTM network, and a U-Net network. The most accurate signal translation was achieved using the U-Net and CNN encoder networks. In the case of the DNN, the results were less accurate but still usable for wrist-to-shoe translation. The CNN-LSTM architecture proved to be unsuitable for such a task due to severe problems with proper signal spike reproduction. The translation to the tibia-worn sensor signals proved more challenging for all tested architectures. This might be caused by less consistent tibia sensor placement and orientation. An efficient translation to the tibia sensor signals might require using more advanced ML models or expanding the training dataset with additional examples.

The results of the performed experiments are promising. The translated results were successfully used to assess the gait parameters of a person walking a 10 m straight path. The differences between the obtained gait and stride durations based on the translated and shoe-worn sensor registered signals were small. The proposed translation algorithm opens an opportunity to use much more common and comfortable wrist-worn sensors (smartwatches or smart bands) and still apply the proven, high-accuracy methods relying on lower limb measurements.

The proposed translation algorithm is a simple one and was used mainly to prove signal translation’s efficiency in gait estimation. There are several areas in which the proposed solution could be improved. First, the translation could be made more accurate by employing other more advanced neural network architectures processing some additional data, e.g., atmospheric pressure. The models could also be modified to output only the selected signals needed by the particular methods instead of all of the components. Additionally, the training efficiency might be improved by using custom loss functions more targeted at the problem. For example, when the method requires signal peaks to be reconstructed with high accuracy, the loss function should focus on that while relaxing the requirements for idle moments. The introduced improvements, however, should be carried out with the algorithm complexity in mind, as excessive complexity might prevent its deployment to wearable devices.

## Figures and Tables

**Table 1:** Parameters of the dense Autoencoder layers.

	Layer	Units	Activation Function
Encoder	Flatten	-	-
	Dense	512	ReLu
	Dense	256	ReLu
	Dense	128	ReLu
Decoder	Dense	256	ReLu
	Dense	512	ReLu
	Dense	1024	sigmoid
	Reshape	-	-

**Table 2:** Parameters of the convolutional autoencoder layers.

	Layer	Filters	kernel Size	Activation Function
Encoder	1D Convolution	64	3	ReLu
	1D MaxPooling	-	2	ReLu
	1D Convolution	128	3	ReLu
	1D MaxPooling	-	2	ReLu
	1D Convolution	256	3	ReLu
Decoder	1D Transp. Convolution	256	3	ReLu
	1D Transp. Convolution	128	3	ReLu
	1D Transp. Convolution	64	3	ReLu
	1D Convolution	6	3	sigmoid

**Table 3:** Parameters of the CNN-LSTM network layers.

Layer	Filters/Units	Kernel Size	Activation Function
1D Convolution	64	3	ReLu
LSTM	64	-	tanh
LSTM	6	-	tanh

**Table 4:** Parameters of the U-Net network.

	Layer	Filters	Kernel Size	Activation Function
Contraction	1D Convolution	64	3	ReLu
	1D MaxPooling	-	2	ReLu
	1D Convolution	128	3	ReLu
	1D MaxPooling	-	2	ReLu
	1D Convolution	256	3	ReLu
Expansion	1D Transp. Convolution	256	3	ReLu
	Concatenate	-	-	-
	1D Transp. Convolution	128	3	ReLu
	Concatenate	-	-	-
	1D Transp. Convolution	64	3	ReLu
	1D Convolution	6	3	sigmoid

**Table 5:** Mean absolute and root mean squared errors for tibia-sensor signal reconstruction. The acceleration *a* and angular velocity ω values are in *g* and degrees per second, respectively.

	Architecture	$a_{x}$	$a_{y}$	$a_{z}$	$\omega_{x}$	$\omega_{y}$	$\omega_{z}$
MAE	DNN	1.0358	0.9056	0.7317	4.1434	3.6224	2.9268
	CNN	0.2980	0.3138	0.3318	1.1919	1.2551	1.3274
	LSTM	0.3826	0.4525	0.5424	1.5303	1.8099	2.1696
	U-Net	0.2840	0.2913	0.3092	1.1358	1.1651	1.2367
RMSE	DNN	1.2142	1.0862	1.0045	4.8566	4.3447	4.0181
	CNN	0.4636	0.4398	0.5037	1.8546	1.7592	2.0146
	LSTM	0.6527	0.6322	0.7800	2.6108	2.5288	3.1202
	U-Net	0.4425	0.4128	0.4765	1.7699	1.6512	1.9062

**Table 6:** Mean absolute and root mean squared errors for shoe-sensor signal reconstruction. The acceleration *a* and angular velocity ω values are in *g* and degrees per second, respectively.

	Architecture	$a_{x}$	$a_{y}$	$a_{z}$	$\omega_{x}$	$\omega_{y}$	$\omega_{z}$
MAE	DNN	0.2541	0.1899	0.2082	1.0165	0.7596	0.8327
	CNN	0.2104	0.2049	0.1901	0.8416	0.8196	0.7602
	LSTM	0.3776	0.2521	0.2854	1.5106	1.0082	1.1415
	U-Net	0.2434	0.2218	0.2105	0.9735	0.8873	0.8418
RMSE	DNN	0.3648	0.2851	0.3034	1.4592	1.1405	1.2134
	CNN	0.3436	0.3446	0.3209	1.3742	1.3785	1.2836
	LSTM	0.7510	0.4759	0.5560	3.0040	1.9035	2.2242
	U-Net	0.4218	0.3907	0.3692	1.6871	1.5629	1.4769

**Table 7:** Temporal gate parameters determined based on shoe-worn sensor measurements and translated results.

Parameter	Shoe Sensor	Translated
mean stride duration [s]	1.136	1.108
mean gait duration [s]	0.568	0.554
mean stance duration [s]	0.573	0.493
mean swing duration [s]	0.564	0.608

## Data Availability

The data used for the study can be found at https://doi.org/10.5281/zenodo.10436579 (accessed on 27 December 2023).

## Abbreviations

The following abbreviations are used in this manuscript:

BP	Blood pressure
CT	Computer tomography
CNN	Convolutional neural network
DNN	Dense neural network
ECG	Electrocardiography
EEG	Electroencephalogram
FC	Fully connected
GEI	Gait energy image
GRNN	Generalized regression neural network
IC	Intrinsic capacity
IMU	Inertial measurement unit
LSTM	Long short-term memory
MAE	Mean average error
ML	Machine learning
MLP	Multi layer perceptron
MR	Magnetic resonance
MSATNet	Multi-scale activity transition network
MSE	Mean squared error
PET	Positron emission-computed tomography
PPG	Photoplethysmogram
SPAE	Stacked progressive autoencoder
UWB	Ultra-wideband
